# Melioidosis in Mexico, Central America, and the Caribbean

**DOI:** 10.3390/tropicalmed3010024

**Published:** 2018-02-26

**Authors:** Javier I. Sanchez-Villamil, Alfredo G. Torres

**Affiliations:** 1 Department of Microbiology and Immunology, University of Texas Medical Branch, Galveston, TX 77555, USA; jaisanch@utmb.edu; 2 Department of Pathology, Sealy Center for Vaccine Development, University of Texas Medical Branch, Galveston, TX 77555, USA

**Keywords:** *Burkholderia pseudomallei*, melioidosis, Mexico, Central America, Caribbean, epidemiology, awareness

## Abstract

*Burkholderia pseudomallei* is the causative agent of melioidosis, an endemic disease in tropical areas around the world. Cumulative human cases have demonstrated that melioidosis is prevalent and increasingly recognized in the American continent. Even though the first reports of melioidosis in Mexico, Central America, and the Caribbean Islands date back to the late 1940s, the potential of the disease as a public health concern in the region has not been fully appreciated. Unfortunately, recent studies predicting the global distribution of the disease and the demonstration of melioidosis endemicity in Puerto Rico have not increased recognition of the disease by health professionals in this region. Furthermore, a lack of both diagnostic capacity and awareness of the disease has resulted in a limited number of studies that have attempted to accurately determine its prevalence and geographical distribution. In this review, a summary of reported cases in the countries of this region are presented, as well as recommendations to increase the diagnosis and awareness of the disease as an important public health problem in Mexico, Central America, and the Caribbean islands.

## 1. Introduction

Melioidosis is an emerging, potentially fatal disease caused by *Burkholderia pseudomallei*, which can be acquired through inoculation, inhalation, or ingestion. Inhalation of the bacterium results in the most rapid and fulminant disease, whereas percutaneous inoculation is slower to progress and is often limited to a cutaneous lesion [1,2]. *B. pseudomallei* can also cause asymptomatic infections in healthy individuals, or can induce an acute, chronic, or latent disease. Melioidosis can be confused with pneumonia or tuberculosis, and the bacterium is resistant to a wide variety of antibiotics, while its pathogenic mechanismsare not completely understood. Further, the disease most commonly affects individuals with underlying conditions, including type 2 diabetes, excessive alcohol consumption, and chronic lung disease [1,3,4]. Target organs commonly include the lung, spleen, and prostate, but *B. pseudomallei* has also been shown to establish infections in the bone marrow, central nervous system, kidneys, and the gastrointestinal tract [5].

The magnitude of melioidosis in the Western Hemisphere is not fully understood. However, cases occur sporadically in the Americas, with an increasing number of them observed among people with no travel history to known endemic countries [6]. New endemic foci have been reported in countries such as Mexico, Costa Rica, Guadeloupe, and Puerto Rico. A prior review of melioidosis cases in the Americas described 120 identified human cases that occurred between 1947 and 2015, 95 of which (79%) were likely acquired in the Americas; the mortality rate was 39% [6], indicating that *B. pseudomallei* is widespread in the American continent. Additionally, Limmathurotsakul et al. predicted that *B. pseudomallei* is present in tropical latitudes, and that the highest risk zones included South and Central America [7]. Mexico had the highest predicted incidence of melioidosis in North America, with 550 cases per 100,000 population each year; while in Central America, El Salvador was predicted to have 114 cases, and, in the Caribbean, 24 cases were predicted for Haiti [7]. In addition to the high prevalence of diabetes (13.1% Mexico, 10.1% Central America, and 11.2% in The Caribbean) [8] and limited access to health care, this study suggested that melioidosis might be endemic in many countries of this region, but be significantly underreported. Given the diagnostic limitations and little or no surveillance, it is likely that *B. pseudomallei* is present in many more tropical countries where it has not yet been identified [9].

Melioidosis requires specific antibiotic treatment, since *B. pseudomallei* has natural resistance to several commonly used antibiotics such as penicillin, ampicillin, first and second generation cephalosporins, gentamicin, tobramycin, and streptomycin [10]. However, it is still important to determine the antimicrobial sensitivity profile to newer antibiotics [11]. Current therapy recommendations are based on the outcome of a number of clinical trials in endemic regions and other clinical observations [12]; however, in non-melioidosis endemic areas, empiric antimicrobial therapy for pneumonia may not include drugs that are active against *B. pseudomallei*. Therefore, melioidosis represents a challenge for clinical and microbiology laboratory staff in regard to diagnosing it accurately, ensuring appropriate therapy, and alerting public health officials to its potential endemicity.

Defining the global distribution and updating the epidemiology of *B. pseudomallei* is important for developing an accurate melioidosis risk map, and expanding the list of countries with sporadic occurrence that may be upgraded to endemic status as a result of the recognition of increasing numbers of indigenous cases and the detection of *B. pseudomallei* in the environment. Further, an accurate disease distribution analysis will help raise awareness among healthcare workers in affected areas. Here, we present an updated review of *B. pseudomallei* cases in this region that were published from 1945 to 2017. We deliberately did not include Canada and the United States of America (USA) as, although there have been two apparent cases of indigenous melioidosis in the continental USA in recent years [13,14], they occurred in a part of the USA that is predicted to not be suitable for the environmental survival of *B. pseudomallei*, and there was no evidence of a local environmental source. We also excluded Canada, since although (similar to the USA) there have been a number of cases imported from other endemic areas [15,16,17,18], there is no concrete evidence of indigenous melioidosis in either country.

## 2. Review of Melioidosis Cases and Presence of *B. pseudomallei* in Each Country

**Figure 1:**
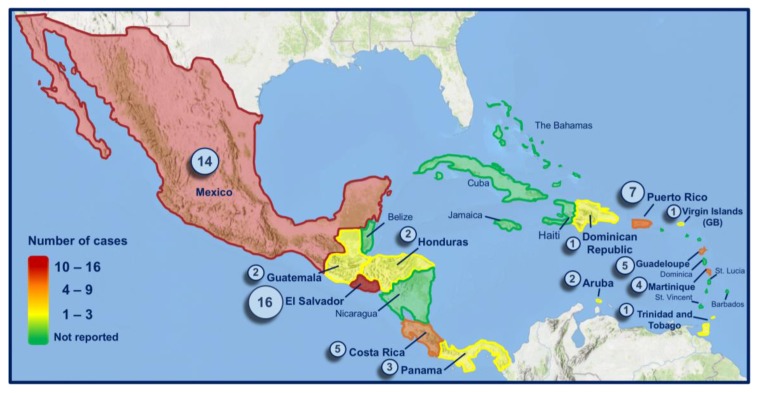
Melioidosis cases reported (1945–2017). Adapted from: Esri, HERE, DeLorme, increment P corp., NPS, NRcan, Ordnance Survey, © OpenStreetMap contributors, USGS, NGA, NASA, CGIAR, N Robinson, NCEAS, NLS, OS, NMA, Geodatastyrelsen, Rijkswaterstaat, GSA, Geoland, FEMA, Intermap, and the GIS user community.

We conducted a search of the literature (PubMed, MEDLINE, and Google Scholar) in order to identify published reports of melioidosis cases originating from Mexico, Central America, and the Caribbean islands. Some keywords used included combinations of ‘*Burkholderia pseudomallei*’, ‘melioidosis’, ‘Mexico’, ‘Central America’, ‘Caribbean’, ‘*Pseudomonas pseudomallei*’, and ‘*Malleomyces pseudomallei*’. Thus, we reviewed 37 papers (from 1945 to 2017) and, when available, the following information was collected from each article: year of diagnosis, number of cases, gender, age, travel history, country of diagnosis, diagnostic laboratory results, signs, symptoms, and patient outcomes. ArcGIS online (ESRI, Redlands, CA, USA) was used to generate the map in Figure 1. Reported cases are summarized in Table 1, and are discussed in more detail below.

### 2.1. Mexico

Fourteen cases of melioidosis have been reported that were likely acquired in Mexico (see Table 1). The first case reported dated from 1958, and was of a 22-year-old white man from Oklahoma, USA, with a travel history to border towns in Mexico [22]. He was diagnosed with melioidosis in the USA by complement fixation studies for ‘*Malleomyces pseudomallei’*. However, no bacteriologic confirmation was obtained on material from the abscesses cavity [22]. The second case, which was diagnosed in the USA, was reported in 1985 [25]. The patient was a 72-year-old Hispanic male, who had lived in the city of Manzanillo all of his life, and who was working as a clerk. He had never traveled outside of Mexico. He died 72 h after admission to the hospital. At autopsy, the lungs showed patchy areas of consolidation, and histological sections revealed suppurative acute bronchopneumonia [25]. The third case was presented in 1989 at a cystic fibrosis conference. Three of 19 sputum samples from patients with cystic fibrosis were positive for ‘*Pseudomonas pseudomallei’*, of which two patients died from pulmonary illness [26]. Years later, in 2009 [40], 2010 [6], and 2013 [40], other cases of melioidosis were reported in Mexico that were diagnosed in USA; however, the documented information regarding signs, symptoms, and travel history is very limited. The only information available from the case reported in 2010 is that it resulted in death [6]. Subsequently, in 2011, two more Mexican patients were diagnosed in the USA, one of whom was a 10-year-old child, and the second was a 22-year-old alcoholic female [6]. In both cases, the patients survived the disease. There is another report of melioidosis diagnosed in Mexico in 2012 in a 29-year-old medical resident with an acute illness (fever, malaise, and dyspnea) with 72 h of disease progression, with a history of recent travel to a tropical Mexican region (Acapulco) one week previously [47]. Computed tomography (CT) showed irregular hyperdense images with ground glass opacities in the lung, and he received treatment with trimethoprim/sulfamethoxazole with adequate progress and cure. Microbiological identification was performed using the VITEK^®^2 (bioMérieux, Marcy-l'Étoile, France) system that has been reported sometimes to have problems differentiating between *B. pseudomallei* and *B. cepacia* [53]. In this case, the isolate was not confirmed by a reference laboratory, such as the Centers for Disease Control and Prevention (CDC). 

Further cases of melioidosis in Mexico have been reported more recently. In 2014, a 59-year-old female with a four-day history of right-sided upper back and anterior chest pain, fever, and shortness of breath was diagnosed in the USA with melioidosis, which was believed to have been contracted in Mexico [50]. She had traveled to Los Cabos, Mexico, one week before hospital admission. A CT scan of the chest showed an irregular mass in the apical segment of the right upper lobe with ground glass opacities, and an enlarged right paratracheal lymph node. The isolate was later confirmed and identified as *B. pseudomallei* by the CDC. It is important to note that the patient had diabetes mellitus and a well-controlled HIV infection, as well as having received a renal transplant. Additionally, she was present when a hurricane hit the zone, and so had multiple risk factors for acquiring the disease. Another important associated risk factor was reported in a 70-year-old smoker from Mexico with no history of travel to any other melioidosis-endemic area [51]. The patient presented with fever, chills, and an enlarging left neck mass. CT revealed a supraclavicular mass that was drained, and the organism grown was confirmed by the CDC as *B. pseudomallei*. The patient reported symptom resolution with a decrease in the size of the supraclavicular mass after antibiotic therapy. In 2015, the first case of melioidosis was reported in a northern state of Mexico (Sonora) in a 48-year-old male who presented with fever and a history of abscess in the right subscapular region with hepatomegaly and splenomegaly, but without pulmonary symptoms [52]. Microbiological identification using the VITEK^®^2 (bioMérieux) system identified the pathogen as *B. pseudomallei*, and the use of trimethoprim/sulfamethoxazole resolved his symptoms. The recent increasing occurrence of melioidosis in patients with no travel history outside Mexico indicates that *B. pseudomallei* is endemic, mainly in tropical regions of the country. It is likely that, due to the lack of advanced diagnostic methods, melioidosis remains an underdiagnosed disease in this country.

### 2.2. Central America

Cases have been reported in Central America from Guatemala to Panama, except in Nicaragua and Belize (Figure 1). 

#### 2.2.1. Guatemala

Only two cases of melioidosis have been reported in Guatemala between 2012–2013 (Table 1). The first report was from a 71-year-old male with diabetes [6], and the second was from a 22-year-old healthy female. In the former case, the potential risk of exposure was the use of a thermal sulfur hot spring in that country [6]. Both cases survived, and were diagnosed in the United States; however, no more detailed information was available.

#### 2.2.2. El Salvador

Thirteen cases of melioidosis were reported in 1981 by Bloch et al. in residents of El Salvador who had never traveled outside of the country [6,23] (Table 1). However, limited information is available from those cases. A subsequent case of melioidosis was reported in 2000 in a 37-year-old female resident of El Salvador who survived the disease, despite having diabetes mellitus [6]. Additionally, Salvadorian refugees have been diagnosed with melioidosis during immigration health screening. One refugee was diagnosed with melioidosis in the USA in 2001 [33]. In this case, the patient presented with a cerebral abscess, but survived. However, the identity of the bacterium was not confirmed by a reference laboratory. The last documented case was diagnosed in 2003 in a 47-year-old male with diabetes mellitus, who had traveled to El Salvador three weeks earlier [35]. A CT scan indicated the presence of pulmonary abscesses, and the patient died from sepsis and multiorgan system failure [35]. Bacterial isolates were confirmed as *B. pseudomallei*. This case is of importance, because the laboratory workers manipulating the samples were exposed to *B. pseudomallei* cultures without utilizing proper laboratory practices; however, no workers were infected. In response to this incident, laboratory safety recommendations for *B. pseudomallei* were revised [35].

#### 2.2.3. Honduras

Two cases of melioidosis were reported in 2005 in patients who traveled to Honduras a few days before hospital admission (Table 1). A 48-year-old male with diabetes and Guillain–Barré syndrome, with a history of recent travel to Honduras, was diagnosed with pneumonia and perirectal abscess. *B. pseudomallei* was isolated from cultures of blood and abscess fluid. Fortunately, the patient survived the infection [36]. In the same year, an 80-year-old female resident of Honduras was admitted to hospital with a diagnosis of pneumonia after four days of headache, fever, and muscle pain [36]. She died after two days in hospital, and *B. pseudomallei* was identified in a blood culture drawn when she was admitted. Both cases were diagnosed in the USA, and the identification of *B. pseudomallei* was confirmed by real-time PCR [36]. 

#### 2.2.4. Costa Rica

Melioidosis in Costa Rica was reported in 1998 in a 56-year-old male with diabetes mellitus, presenting with fever and lung infection; he died a few days after hospital admission [30] (Table 1). The isolate from blood and bronchial aspirate was later confirmed and identified as *B. pseudomallei* by VITEK^®^2 (bioMérieux) [30]. The second case diagnosed was reported in 2000 in a 63-year-old male smoker with diabetes mellitus and a history of working with cattle, [6,32]. The patient developed fever and a cough with yellowish expectoration. A chest roentgenogram revealed an inflammatory infiltrate in the right lung. He died 72 h post-admission. *B. pseudomallei* was identified from a blood sample and bronchial secretions by the National Reference Laboratory (Hospital Nacional de Niños, Costa Rica). Another case was reported in 2009, but no detailed information was provided [41]. In 2010, a 42-year-old female was diagnosed with melioidosis, which was probably acquired while vacationing in Costa Rica [6]. The latter two patients both survived. In 2014, the first case of *B. pseudomallei* infection of the central nervous system was reported in this country [49]. A 45-year-old male with diabetes mellitus presented with headache, fever, photophobia and convulsive crisis. Cerebrospinal fluid and empyema cultures were positive for *B. pseudomallei*, as identified by the VITEK^®^2 (bioMérieux) system and confirmed by a local reference laboratory (INCIENSA) [49]. The patient survived after 20 weeks of ceftazidime and trimethoprim/sulfamethoxazole treatment.

#### 2.2.5. Panama

The first reports of melioidosis in the Americas, dating from 1945–1957, were acquired in Panama (Table 1). The first of these possible cases of melioidosis was diagnosed in the USA in 1945 in a 31-year-old male who was employed in the Panama Canal Zone from 1927–1928 [19]. The patient was admitted to hospital for the diagnosis and treatment of sinuses and ulcers of the right buttock and thigh that had been present for eight years [19]. The organism was isolated from pus and tissue debris, and was reported as ‘*Malleomyces pseudomallei’*. It was reported that before the chronic phase of the disease, the patient had suffered from an acute pneumonic phase. A second case of melioidosis, which was probably acquired in Panama, was reported in 1957 [20,21]. The patient was a 20-year-old male who was treated for ‘flu-like syndrome’. After three days, he developed genitourinary symptoms, and later became systemically ill. He had pyuria, and a chest roentgenogram showed a small infiltrate in the upper lobe of the left lung. After one month, he developed an acute septic arthritis of the shoulder joint; pus was removed, and *Pseudomonas pseudomallei* was isolated in culture [20]. However, in the two aforementioned cases, the cultures were not available for further studies, and could not be considered as verified cases of melioidosis. Another case of melioidosis in Panama has been reported more recently. In 2011, a 31-year-old male with diabetes mellitus, who developed liver and skin abscesses, was diagnosed with *B. pseudomallei* infection [46]. The patient had no travel history outside the country [46]. Unfortunately, limited information about the methodology that was used for *B. pseudomallei* identification was included.

### 2.3. Caribbean Islands

#### 2.3.1. Puerto Rico

As of October 2017, there have been seven cases of melioidosis reported or associated with Puerto Rico, based on case reports or submissions to the USA CDC reference laboratory. The earliest case was reported in 1982 in a 62-year-old Puerto Rican woman with systemic lupus erythematosus [24] (Table 1). The patient had a one-week history of fever, chills, generalized weakness, and malaise. She had a history of chronic non-productive cough; additionally, she had never traveled outside the country. The patient died 48 h after admission, and the CDC later confirmed the organism as *Pseudomonas pseudomallei* [24]. The second case reported in Puerto Rico was in 1998 in an 11-year-old male with chronic granulomatous disease [28]. At the time of admission, he presented with fever, right neck pain, and two weeks of productive cough. A chest CT revealed a right hilar mass and right paraesophageal lymphadenopathy, as well as a right supraclavicular mass. The API rapid NFT (non-fermentative) system identified *B. pseudomallei*, and this identification was subsequently confirmed by the CDC. The patient died after a few days of antibiotic therapy [28]. In 2003, a *B. pseudomallei* infection was reported in a 55-year-old female with diabetes mellitus, who had been exposed to flood waters during the rainy season in this country [34]. This report highlighted the role played by flood waters as a risk factor in the epidemiology of melioidosis. In 2009, an 88-year-old male Puerto Rican veteran who had served in Korea and Panama was diagnosed in USA with melioidosis [6,40]. The potential risk of exposure was environmental (digging a ditch), and he survived the disease [6,40]. No additional information was provided. In 2010, a 38-year-old male presented with symptoms of chest pain and shortness of breath; his only relevant medical history was obesity [42]. He died 1 h after arriving at the hospital. A histological analysis demonstrated acute necrotizing pneumonia with pyogranulomatous splenitis, hepatitis, and focal myocarditis. *B. pseudomallei* was identified by the CDC [42]. Doker et al. also reported another confirmed case of melioidosis in 2012 [42]. A 60-year-old male with a three-day history of nausea, anorexia, and abdominal and chest pain, was admitteded to an emergency room [42]. He had diabetes mellitus, obesity, alcohol use, and was a smoker.In addition, the patient reported intermittent intravenous use of heroin and cocaine. *B. pseudomallei* was identified and confirmed at the CDC; the patient survived [42]. Thus, the earliest case in Puerto Rico was reported in 1982, followed by one in each of the following years: 1998, 2003, 2009, 2010, 2012, and 2017. Six of the seven clinical cases came from the eastern portion of the island. No information is currently available on the origins of the seventh case, which occurred in the summer of 2017. 

Due to the sporadic nature of these reports, surveillance was not considered a priority until the 2012 case, which prompted a closer look at the potential local endemicity of the disease [42]. The study included a serological survey, which found that 6% of the contacts of the 2010 patient and 25% of the contacts of the 2012 patient were seropositive; the study also found an association between seropositivity and a history of skin wounds and/or a history of drug use. Environmental sampling of soil near the 2012 patient’s residence yielded two *B. pseudomallei* isolates that were closely related to the isolate from the patient, strongly indicating that this area was the source of his infection [42,54]. The results of this study prompted an extensive environmental survey of soil and water based on potential habitats around the island. Isolates of *B. pseudomallei* were recovered from soil samples in the northern part, indicating that the organism is not geographically restricted to the eastern portion of the island (C.M. Hall, personal communication). This suggests that surveillance around the island may yield more cases. However, the effects of Hurricane Maria in 2017 were still being dealt with at the time of writing this manuscript. It is unknown how long it will take to rebuild the public health infrastructure of the island.

In addition to the presence of *B. pseudomallei* in humans and the environment in Puerto Rico, Hemme et al. found the presence of antibodies against *B. pseudomallei* in one of 24 non-human primates (*Erythrocebus patas*) that were tested [55]. This study suggested probable transmission through contact with contaminated soil and water.

#### 2.3.2. Other Caribbean Islands

Sporadic cases of melioidosis have been associated with other Caribbean islands, and are typically diagnosed in tourists who return to their home countries or residents who seek medical treatment or consultation in countries with laboratory capability to diagnose the disease. Islands that are associated with cases of melioidosis include Aruba [39,45], the British Virgin Islands [37], Guadeloupe [29,43,56], the Dominican Republic [38], Martinique [27,31,44], Trinidad, and Tobago [18]. A case of melioidosis was reported in a child with cystic fibrosis who had travelled to Aruba some three months previously [39]. Interestingly, the occurrence of melioidosis in animals, particularly sheep, goats, and pigs, had previously been reported in the 1950s [57]. Unfortunately, no isolates from the animal melioidosis outbreak were available for confirmation; however, this remains the only reported instance of animal meliodosis in this region.Two cases of melioidosis have been reported from Guadeloupe [29,43] and a report of three further cases from Guadeloupe in 2017 is also in submission [56]. The CDC is also currently investigating two cases of melioidosis in the US Virgin Islands [56]. Based on predicted environmental suitability, it is likely that other Caribbean islands may be endemic for this bacterium [7]. Discussions with various stakeholders in the Caribbean indicate that surveillance for melioidosis is typically not a priority for them [56].

## 3. Current Challenges in the Diagnosis and Treatment ofMelioidosis in the Region

Melioidosis has generated further interest in view of the detection of *B. pseudomallei* infection in countries that were previously considered non-endemic (Figure 1). These sporadic occurrences over a prolonged period are unlikely to have resulted from repeated importation, and along with a high predicted incidence of the disease in this region [7], suggest that the pathogen is likely to be endemic but underdiagnosed across this region. The high proportion of melioidosis cases in this review that were diagnosed and confirmed outside of the region, mainly in the USA (26 of 63 cases, representing 41.27%), strongly suggests a lack of reliable diagnostic testing within the region. Significant confusion exists regarding the identification and diagnosis of melioidosis in this part of the world. Puerto Rico was the first country in the region where *B. pseudomallei* endemicity was clearly demonstrated [42].However, in Mexico, Central America, and other Caribbean islands, the diagnosis is being made with increasing frequency based on identification with commercial systems such as API 20E, API 20NE, VITEK^®^1 and 2 (bioMérieux). However, a limitation of these commercially-available systems is that the misidentification of *B. pseudomallei* for *B. cepacia* may occur with VITEK^®^2 [53]. However, API 20NE and 20E have correctly identified 98% and 99% of *B. pseudomallei* cases, respectively [58].

There is a variety of serological assays (ELISAs, indirect hemagglutination assay, and indirect immunofluorescence assay) available for the identification of *B. pseudomallei* in endemic regions, although their usefulness is limited by high rates of background antibody [59,60]. However, seroprevalence studies would be very valuable to assess the endemicity in this region, because only Puerto Rico has been clearly demonstrated as endemic, using a combined approach of seroepidemiology and environmental detection [42]. Ashdown’s selective agar is still considered the gold standard for optimal diagnosis, and is recommended for the isolation of *B. pseudomallei* from clinical specimens from sites with a normal flora. However, it is not always available and is not routinely used in non-endemic areas [61]. *B. pseudomallei* grows on many commercially available culture media (MacConkey, sheep blood and chocolate agar), but it is likely that many laboratories in the region may misidentify the bacterium as *Pseudomonas* species or other *Burkholderia* species, particularly with some commercial systems with poorly ability to identify *B. pseudomallei* [62,63,64]. Likewise, clinical manifestations of melioidosis do not make the diagnosis any simpler, because the disease is frequently clinically indistinguishable from many infections caused by other bacteria. This indicates that a limited experience and lack of familiarity with *B. pseudomallei* amongst clinicians and laboratory staff, in combination with unreliable diagnostic tests in non-endemic regions, may result in continued misidentification of the pathogen, and therefore underestimation of melioidosis cases in the region.

Pneumonia was the most common clinical presentation associated with the melioidosis cases described here. Chronic melioidosis with cavitary lung lesions and subcutaneous and visceral abscesses were also found in many cases, and the protean nature of melioidosis was evidenced by one case affecting the central nervous system. Therefore, the diagnosis of melioidosis should be considered by physicians in any patients presenting with those signs and symptoms who are returning from or residing in potentially endemic tropical regions. In addition, medical personnel should be aware of the conditions that predispose to melioidosis, including diabetes mellitus, alcoholism, chronic lung disease, cancer, and other immunosuppressive disorders [62], which should increase the chances of making the diagnosis.

The accurate diagnosis of melioidosis is important in order to guide antibiotic treatment and prevent relapse. The recent recommendations for therapy of melioidosis have been comprehensively summarized elsewhere [12,65]. One other issue requiring attention to reduce the risk of melioidosis acquisition in the healthcare setting is the possible accidental exposure of laboratory workers to infection. Sniffing culture plates is an unsafe laboratory procedure that is still performed in some countries of this region, and should be forbidden. Based on anecdotal experience, several clinical laboratorians in Mexico routinely sniff culture plates in order to identify the characteristic smell (referred to as a corn ‘tortilla’-like) of *Pseudomonas aeruginosa* colonies (with which *Burkholderia* spp. may be confused), which is a potentially high-risk activity in a laboratory setting. Consequently, physicians should notify laboratory staff when samples are submitted from patients with symptoms, risk factors, or history that is suggestive of melioidosis.

## 4. Awareness of Melioidosis

Disease awareness and education are important for melioidosis prevention and diagnosis. Consequently, physicians and health care personnel must be made aware of the increasing number of cases of apparently indigenous melioidosis reported in Mexico, Central America, and the Caribbean islands. For example, health departments should raise awareness of melioidosis by distributing printed information, in addition to running training seminars and workshops for healthcare professionals. The future development of practical tools to detect, assess, and notify cases should be evaluated and implemented by health organizations such as PAHO (Pan American Health Organization) and CARPHA (The Caribbean Public Health Agency); it is worth noting that the websites for these agencies do not currently mention melioidosis. Modeling of the predicted distribution and burden of melioidosis showed that the population at risk in this region is about 246 million, in which 2000 could have melioidosis, and 1000 might die each year [7]. If the prediction is correct, melioidosis is more than a sporadic disease in this region.

Unfortunately, public awareness of melioidosis is very low, even in endemic countries such as Thailand [66]. It is evident that people from Mexico, Central America, and the Caribbean Islands are not aware of the disease, and therefore, they do not know anything about how to prevent it. Raising awareness and knowledge about melioidosis among researchers and representatives of the public health sectors will allow the development and implementation of guidelines for prevention, diagnosis, and therapy. Furthermore, health departments should consider making melioidosis a reportable disease, which would help improve surveillance and encourage the redefinition of endemic areas.

## 5. Future Challenges

Current melioidosis morbidity and mortality in well-known endemic regions have decreased due to better clinical management and advances in diagnostic techniques. However, in Mexico, Central America, and the Caribbean islands, the inadequate diagnosis and treatment of patients with melioidosis is a public health problem that is almost certainly leading to unnecessary deaths. In this context, national reporting systems should be improved in order to inform strategies to prevent, prepare for, detect, and respond to melioidosis cases.

Therefore, (1) timely and accurate diagnosis is needed to ensure effective antibiotic therapy; (2) there is a need to introduce selective media, such as Ashdown agar, in order to improve the yield of *B. pseudomallei* from patients who are suspected to have melioidosis; (3) it is necessary to instruct healthcare professionals about diagnosis, treatment, and preventive measures to combat melioidosis; (4) reporting cases that occur in areas where melioidosis is not currently recognized as endemic will help technicians and clinicians become familiar with this pathogen, and alert public health officials. Finally, recognizing melioidosis as an endemic disease affecting this region will allow the establishment of educational campaigns for professionals and the general public, especially those with risk factors, and the development of guidelines for the prevention and treatment of the disease.

## Figures and Tables

**Table 1:** Summary of published melioidosis cases in Mexico, Central America, and the Caribbean.

Year	Number of Cases	Patient (Age and Gender)	Country Where Infection Likely Occurred	Travel History	Country of Diagnosis	Outcome	Ref.
1945 ^a^	1	31-year-old male	Panama	Panama	USA	Discharged	[19]
1957 ^b^	1	20-year-old male	Panama	Panama	USA	Survived	[20,21]
1958 ^a^	1	22-year-old male	Mexico	Mexico, Japan, and Korea	USA	Survived	[22]
Unknown	13	Variable	El Salvador	Residents of El Salvador with unknown travel history	El Salvador	10 survived/3 died	[6,23]
1982 ^b^	1	62-year-old female	Puerto Rico	NR	Puerto Rico	Died	[24]
1985 ^b^	1	72-year-old male	Mexico	No travel history outside Mexico	USA	Died	[25]
1989	3	Variable	Mexico	Unknown	Mexico	1 survived/2 died	[26]
1994 ^c^	1	66-year-old male	Martinique	Travel history to Africa and South America	France	Survived	[27]
1997	1	11-year-old male	Puerto Rico	Travel history to USA for medical care	USA	Died	[28]
1997	1	4-year-old female	Guadeloupe	Resident of France with travel history to Guadeloupe	Guadeloupe	Survived	[29]
1998	1	56-year-old male	Costa Rica	Unknown	Costa Rica	Died	[30]
1998	1	Age of female unknown	Martinique	Unknown travel history	France	Survived	[31]
1999	1	Age of male of unknown	Martinique	Unknown travel history	France	Survived	[31]
2000	1	63-year-old male	Costa Rica	No travel history outside Costa Rica	Costa Rica	Died	[6,32]
2000	1	37-year-old female	El Salvador	Resident of El Salvador	USA	Survived	[6]
2001	1	Unknown age or sex	El Salvador	Resident of El Salvador	USA	Survived	[33]
2003	1	55-year-old female	Puerto Rico	Resident of Puerto Rico with travel to USA	Puerto Rico	Died	[34]
2003	1	47-year-old male	El Salvador	Travel history to El Salvador	USA	Died	[6,35]
2005	2	48-year-old male80-year-old female	Honduras	Travel history to Honduras	USA	1 survived/1 died	[36]
2006	1	17-year-old male	British Virgin Islands	British Islands and Canada	Canada	Survived	[37]
2009	1	17-year-old male	Dominican Republic	Resident of Dominican Republic with travel history to Argentina	Argentina	Survived	[38]
2009	1	7-year-old female	Aruba	Travel history to Puerto Rico, Portugal, and Australia	USA	Survived	[39]
2009	1	88-year-old male	Puerto Rico	Veteran with service in Panama and Korea	USA	Survived	[6,40]
2009	1	Unknown	Costa Rica	Unknown	USA	Survived	[41]
2009	1	Unknown	Mexico	Unknown	USA	Unknown	[40]
2010	1	30-year-old male	Mexico	Resident of Mexico	USA	Died	[6,40]
2010	1	38-year-old male	Puerto Rico	No travel history outside of Puerto Rico	Puerto Rico	Died	[42]
2010	1	15-year-old female	Guadeloupe	Resident of France with travel history to Guadeloupe	France	Survived	[40,43]
2010	1	35-year-old male	Martinique	Resident of Switzerland with travel history to Martinique	Switzerland	Died	[44]
2010	1	42-year-old female	Costa Rica	Costa Rica and Mexico	USA	Survived	[6]
2011	2	22-year-old male 10-year-old female	Mexico	Travel history to Mexico	USA	2 survived	[6]
2011	1	46-year-old female	Aruba	Resident of UK with travel history to Aruba and the Caribbean	USA	Survived	[45]
2011	1	31-year-old male	Panama	No travel history outside Panama	Panama	Survived	[46]
2012	1	60-year-old male	Puerto Rico	Resident of Puerto Rico	USA	Survived	[40,42]
2012	1	71-year-old male	Guatemala	Resident of Guatemala	USA	Survived	[6,40]
2012	1	29-year-old male	Mexico	Travel to Acapulco, Mexico one week before disease	Mexico	Survived	[47]
2013	1	22-year-old female	Guatemala	Resident of Guatemala	USA	Survived	[6]
2013	1	66-year-old male	Mexico	Served in Vietnam war	USA	Survived	[40,48]
2014	1	45-year-old male	Costa Rica	Unknown travel history	Costa Rica	Survived	[49]
2014	1	59-year-old female	Mexico	Travel to Los Cabos, Mexico 7 days before disease	USA	Survived	[50]
2014	1	70-year-old female	Mexico	No travel history outside Mexico	USA	Survived	[51]
2014	1	17-year-old male	Trinidad and Tobago	Travel history to Trinidad and Tobago	Canada	Survived	[18]
2015	1	48-year-old male	Mexico	No travel history outside Mexico	Mexico	Survived	[52]
2017	3	Unknown	Guadeloupe	Unknown	Guadeloupe	2 survived/1 died	[41]
2017	1	Unknown	Puerto Rico	Puerto Rico	USA	Survived	Unpublished data from CDC

^a^ The bacterial culture was reported as *Malleomyces pseudomallei*. ^b^ The bacterial culture was reported as *Pseudomonas pseudomallei*. ^c^ Bacterial cultures after this year were reported as *Burkholderia pseudomallei*. NR, not reported.
